# Systemic inflammation response index (SIRI) on the 3rd postoperative day are associated with severe pneumonia in cerebral hemorrhage patients: A single-center retrospective study

**DOI:** 10.1097/MD.0000000000035587

**Published:** 2023-10-27

**Authors:** Yongfeng Zhao, Xian Wang, Hongbo Ren, Yuan Yao

**Affiliations:** a Department of Hematology, The First Affiliated Hospital of Yangtze University, Jingzhou, China; b Department of Pharmacy, The First Affiliated Hospital of Yangtze University, Jingzhou, China; c Department of Neurosurgery, The First Affiliated Hospital of Yangtze University, Jingzhou, China.

**Keywords:** cerebral hemorrhage, inflammatory response, neutrophil-lymphocyte ratio, platelet-lymphocyte ratio, system inflammation response index, systemic immune inflammation index

## Abstract

Inflammatory response was involved in the progression of cerebral hemorrhage. We sought to explore the associations of easily obtained inflammatory indicators including blood cell counts and the ratios of different blood cells counts with pneumonia and severe pneumonia in cerebral hemorrhage patients. We carried 1 retrospective study including 200 patients with cerebral hemorrhage and surgeries. The associations of neutrophils, lymphocytes, monocytes, platelets, systemic immune inflammation index (SII), systemic inflammation response index (SIRI), neutrophil-lymphocyte ratio (NLR), and platelet-lymphocyte ratio (PLR) with pneumonia and severe pneumonia in cerebral hemorrhage patients were estimated by univariate analysis and multivariate logistic regression model. Among the 200 patients included, there were a total of 163 (81.5%) had pneumonia after surgeries. Among 163 cerebral hemorrhage patients with pneumonia, 60 (36.8%) cases were evaluated as severe pneumonia. The level of SIRI on the 1st postoperative day in patients with severe pneumonia was higher than non-severe pneumonia (10.89 ± 12.10 × 10^9^/L vs 7.14 ± 9.76 × 10^9^/L, *P* = .003). The level of SIRI on the 3rd postoperative day in patients with severe pneumonia was more significantly higher (7.98 ± 7.46 × 10^9^/L vs 4.10 ± 3.74 × 10^9^/L, *P* < .001). The results of multivariate analysis showed that SIRI level on the 3rd postoperative day (>6.5 × 10^9^/L) was associated with severe pneumonia in cerebral hemorrhage patients (OR: 4.409, 95% CI: 1.799–10.806, *P* = .001). SIRI was possibly a superior predictor for severe pneumonia in cerebral hemorrhage patients compared with other inflammatory indicators. On the one hand, we intend to validate the cutoff value of SIRI for predicting severe pneumonia in larger samples and multicenter studies. On the other hand, we also intend to use this index to guide the choice of antibacterial drugs in order to better benefit patients.

## 1. Introduction

Cerebral hemorrhage was a common type of stroke. Cerebral hemorrhage accounted for approximately 50% of stroke.^[1,2]^ A survey of 480,687 participants from 155 urban and rural centers in 31 provinces of China showed that the incidence of spontaneous intracranial hemorrhage was 23.8%.^[3]^ The mortality of cerebral hemorrhage was possibly 40% after 30 days of onset.^[4]^ Only about 20% of patients could recover and take care of themselves after 6 months,^[5]^ bringing a heavy burden to society and families. Pneumonia was one common complication of cerebral hemorrhage. The incidence of pneumonia in elderly patients with cerebral hemorrhage was 25.6%.^[6]^ In the intensive care unit, 42% of patients with intracerebral hemorrhage were diagnosed with ventilator associated pneumonia.^[7]^ There were about 66% of patients with cerebral hemorrhage developing hospital acquired pneumonia between 2nd to 5th days during hospitalization.^[8]^ Concomitant pneumonia possibly affected the outcomes of patients with cerebral hemorrhage, the mortality rate could reach 18%.^[7]^

In recent years, some studies have explored the associations between inflammatory factors and concomitant pneumonia in cerebral hemorrhage patients. In the study by Dzhulaĭ GS et al, the results showed that interleukin-1 alpha level both in serum and cerebrospinal fluid increased in the presence of nosocomial pneumonia since the first day of stroke. Interleukin-1 alpha level could be an early risk factor of poor outcome in patients with cerebral hemorrhage.^[8]^ Among 329 patients with cerebral hemorrhage included in the analysis by Alsumrain M. et al, there were 183 (55.6%) cases developing pneumonia. The results of univariate and multivariate logistic regression showed that levels of NLR and PLR at admission were independent predictors of pneumonia.^[5]^ There were also studies showing that SIRI at admission was associated with pneumonia in cerebral hemorrhage patients.^[9]^ The analysis of receiver operating characteristic curve (ROC) in 1 study showed that NLR at admission was the best predictor of stroke associated pneumonia compared with other inflammatory indicators.^[10]^ However, there were few studies focusing on the associations between postoperative inflammatory indicators with concomitant pneumonia or severe pneumonia in cerebral hemorrhage patients with surgeries, which was the objective of our study.

## 2. Materials and Methods

### 2.1. Patients

**Figure 1.**
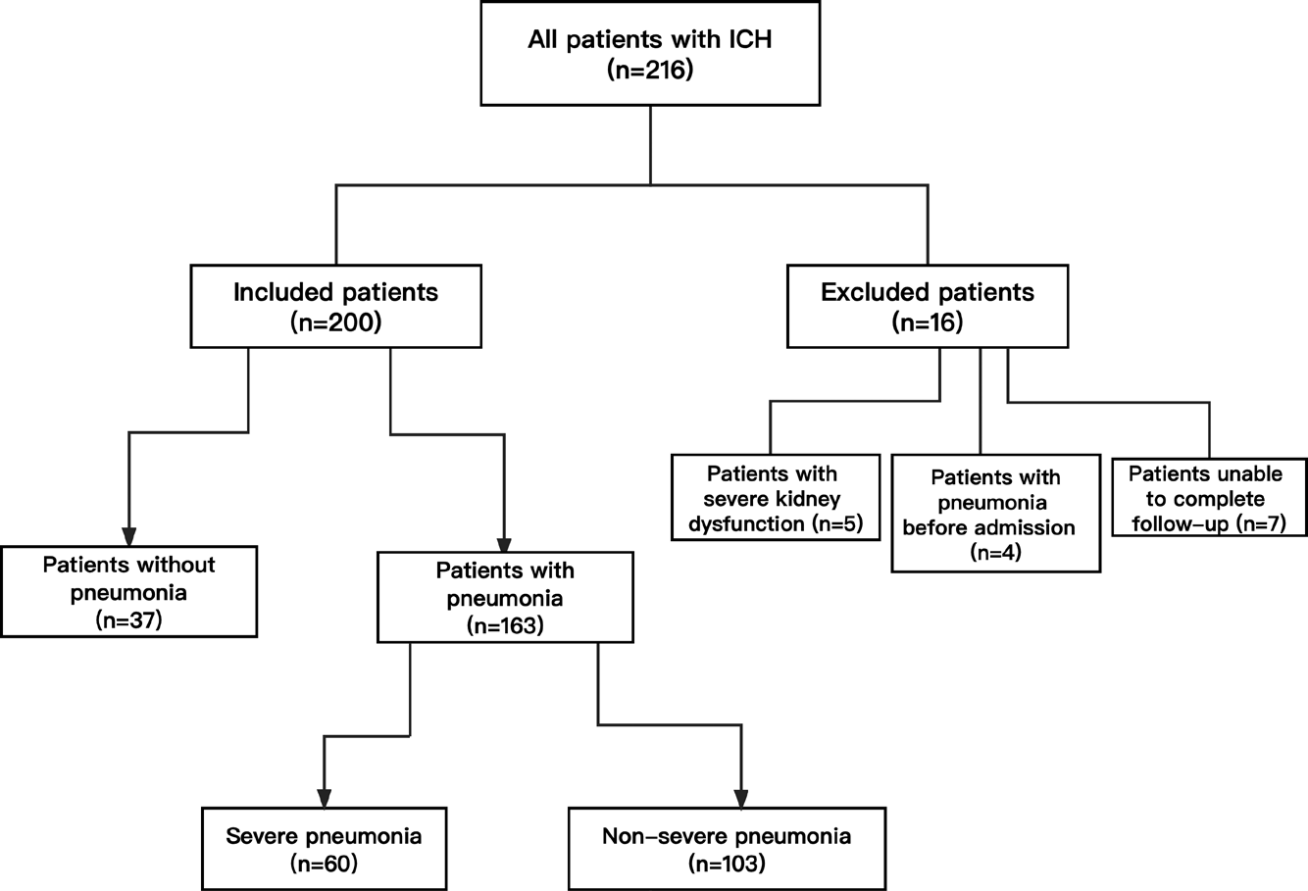
The flow chart of the study. Among all included patients, a total of 163 (81.5%) had pneumonia after surgeries. Among 163 patients with pneumonia, 60 (36.8%) cases were evaluated as severe pneumonia.

One retrospective study was conducted in the department of neurosurgery, the First Affiliated Hospital of Yangtze University from January 2019 to April 2023. Our study complied with the requirements of the Ethics Committee. In addition, the study was reported in line with the STROBE guidelines and was conducted in accordance with the Declaration of Helsinki. Patients were included if they met the following criteria: aged ≥ 18 years; having cerebral hemorrhage confirmed by computed tomography (CT); admitted 48 hours after onset and undergoing surgeries within 48 hours after admission. Patients were excluded if they met the following criteria: dead within 48 hours of admission; diagnosed with pneumonia by CT before admission; diagnosed with subarachnoid hemorrhage by CT; diagnosed with hemorrhage after ischemic stroke; with hematologic diseases; with severe renal dysfunction, liver dysfunction, and immunosuppression. The flowchart was showed in Figure 1.

### 2.2. Data collection

The following baseline characteristics were collected from all included patients: demographic characteristics, combination of lung diseases (emphysema or pulmonary bulla, bronchiectasis, rib fracture, etc), score of Glasgow coma scale (GCS) at admission (a lower score indicating a poor level of consciousness), combination of ventricular hemorrhage at admission and surgical duration.

Laboratory data including neutrophil count, lymphocyte count, monocyte count, and platelet count at 3 time points (preoperative, the 1st postoperative, and the 3rd postoperative days) were collected and analyzed, which was completed in the laboratory of hospital. Counts of blood cells on these days were obtained by flow cytometry (Sysmex XN-9000 Blood Analyzer, Japan). We calculated NLR, PLR, SII and SIRI based on the above blood test results. NLR = neutrophil count/lymphocyte count; PLR = platelet count/lymphocyte count; SII = (neutrophil count × platelet count)/lymphocyte count; and SIRI = (neutrophil count × monocyte count)/lymphocyte count.

We referred to the diagnostic and treatment guidelines for hospital-acquired pneumonia in adult in China for diagnosis. The diagnosis of pneumonia and severe pneumonia in patients required 2 experienced physicians to jointly evaluate. The patient was diagnosed with pneumonia or severe pneumonia according to respiratory symptoms, signs, chest CT or X-ray results, and laboratory examinations, but also met the following conditions. The results of chest CT or X-ray showed new or progressive infiltrative shadows, consolidation shadows, or ground glass shadows, plus 2 or more of the following criteria: fever (body temperature ≥ 38˚C); purulent airway secretions; white blood cell count ≥ 10 × 10^9^/L or < 4 × 10^9^/L. If the patient needed tracheal intubation for mechanical ventilation, or had septic shock, he would be diagnosed as severe pneumonia. Other secondary criteria for severe pneumonia included: respiratory rate ≥30 per minute, oxygenation index ≤250 mm Hg, multiple lobar infiltration, disorders of consciousness, disorientation, blood urea nitrogen ≥7.14 mmol/L, systolic blood pressure <90 mm Hg.^[11–13]^

### 2.3. Statistical analysis

SPSS 23.0 (IBM SPSS Inc., Chicago, USA) was used for data analysis. The qualitative variables were compared by Pearson chi-square test, Continuity correction or Fisher exact test. If the quantitative variables met the normal distribution and variances were homogeneous, independent-sample t-test was used for analysis. If the quantitative variables met the normal distribution but variances were not homogeneous, corrected t-test was used. If the normal distribution could not be satisfied, Mann–Whitney *U* test was used for analysis. The multivariate logistic regression model was constructed for factors with pneumonia or severe pneumonia in cerebral hemorrhage patients. Covariates that had a *P* value <.05 in the univariate analysis were added to the multivariate logistic analysis. The receiver operating characteristic curve (ROC) was used for the cutoff value analysis. All *P* values were 2-sided and the statistical significance was set at *P <* .05.

## 3. Results

### 3.1. Baseline characteristics

Among the 200 patients included, 158 were males and 42 were females. The median age was 61 years. There were 56 patients with lung diseases, including 13 cases with rib fracture, 8 cases with bronchiectasis and 35 cases with emphysema. There were 65 cases with GCS score <11, and 48 cases with intraventricular hemorrhage (Table 1). The median surgical duration was 115 minutes. Among all patients, the preoperative NLR level was 7.29 ± 10.15, PLR level was 165.63 ± 141.33, SIRI level was 4.05 ± 6.27 × 10^9^/L, and SII level was 1254.27 ± 1895.80 × 10^9^/L (Table 2).

**Table 1:** Baseline clinical characteristics in all patients.

	All patients (n = 200)	Without pneumonia (n = 37)	Pneumonia (n = 163)	*P* value
Gender [n (%)]				
Male	158 (79.0%)	29 (78.4%)	129 (79.1%)	.918
Female	42 (21.0%)	8 (21.6%)	34 (20.9%)	
Age (yr)	61 ± 16	60.51 ± 13.08	59.71 ± 13.25	.739
Lung diseases [n (%)]				
No	144 (72.0%)	29 (78.4%)	115 (70.6%)	.338
Yes	56 (28.0%)	8 (21.6%)	48 (29.4%)	
Surgical duration (min)	115 ± 115	90 ± 73	122.50 ± 120	.013
Glasgow coma scale score [n (%)]				
<11	65 (32.5%)	6 (16.2%)	59 (36.2%)	.019
≥11	135 (67.5%)	31 (83.8%)	104 (63.8%)	
With intraventricular hemorrhage [n (%)]				
No	152 (76.0%)	36 (97.3%)	116 (71.2%)	.002
Yes	48 (24.0%)	1 (2.7%)	47 (28.8%)	
		Severe pneumonia (n = 60)	Non-severe pneumonia (n = 103)	*P* value
Gender [n (%)]				
Male		48 (80.0%)	81 (78.6%)	.837
Female		12 (20.0%)	22 (21.4%)	
Age (yr)		64 ± 17	59 ± 16	.339
Lung diseases [n (%)]				
No		41 (68.3%)	74 (71.8%)	.635
Yes		19 (31.7%)	29 (28.2%)	
Surgical duration (min)		160 ± 110	100 ± 90	<.001
Glasgow coma scale score [n (%)]				
<11		42 (70.0%)	17 (16.5%)	<.001
≥11		18 (30.0%)	86 (83.5%)	
With intraventricular hemorrhage [n (%)]				
No		39 (65.0%)	77 (74.8%)	.185
Yes		21 (35.0%)	26 (25.2%)	

**Table 2:** Levels of inflammatory indicators in patients with pneumonia and without pneumonia.

	All patients (n = 200)	Without pneumonia (n = 37)	Pneumonia (n = 163)	*P* value
On the preoperative day				
Neutrophils (×10^9^/L)	7.69 ± 6.00	4.95 ± 3.34	8.27 ± 5.80	<.001
Lymphocytes (×10^9^/L)	1.01 ± 0.85	1.02 ± 0.51	0.99 ± 0.94	.393
Monocytes (×10^9^/L)	0.54 ± 0.35	0.42 ± 0.20	0.56 ± 0.35	.014
Platelets (×10^9^/L)	182.58 ± 69.29	177.94 ± 53.96	183.61 ± 72.34	.658
SIRI (×10^9^/L)	4.05 ± 6.27	2.10 ± 2.61	4.57 ± 6.99	<.001
SII (×10^9^/L)	1254.27 ± 1895.80	732.88 ± 571.87	1451.55 ± 1973.28	.001
NLR	7.29 ± 10.15	4.62 ± 3.78	8.77 ± 11.66	.001
PLR	165.63 ± 141.33	160.67 ± 82.91	174.53 ± 157.13	.475
On the 1st postoperative day				
Neutrophils (×10^9^/L)	9.20 ± 4.14	8.32 ± 3.91	9.40 ± 4.24	.053
Lymphocytes (×10^9^/L)	0.78 ± 0.45	0.84 ± 0.48	0.78 ± 0.47	.320
Monocytes (×10^9^/L)	0.69 ± 0.46	0.73 ± 0.47	0.67 ± 0.46	.477
Platelets (×10^9^/L)	159.52 ± 64.69	165.81 ± 75.77	158.43 ± 62.80	.593
SIRI (×10^9^/L)	7.82 ± 9.97	6.91 ± 6.16	8.55 ± 10.84	.075
SII (×10^9^/L)	1741.64 ± 1548.52	1618.72 ± 967.57	1805.97 ± 1731.79	.179
NLR	12.59 ± 9.29	10.26 ± 4.38	13.12 ± 9.92	.039
PLR	188.34 ± 148.23	187.78 ± 125.06	189.80 ± 149.73	.642
On the 3rd postoperative day				
Neutrophils (×10^9^/L)	7.26 ± 3.06	5.25 ± 2.44	7.39 ± 3.89	.007
Lymphocytes (×10^9^/L)	1.08 ± 0.62	1.33 ± 0.56	1.07 ± 0.62	.038
Monocytes (×10^9^/L)	0.76 ± 0.39	0.75 ± 0.48	0.76 ± 0.41	.332
Platelets (×10^9^/L)	197.50 ± 102.25	178.05 ± 54.11	201.90 ± 87.69	.110
SIRI (×10^9^/L)	4.77 ± 6.25	3.27 ± 3.24	5.28 ± 7.09	.003
SII (×10^9^/L)	1181.57 ± 1098.44	737.52 ± 458.34	1294.18 ± 1110.95	<.001
NLR	6.18 ± 5.65	4.36 ± 2.36	6.83 ± 6.09	<.001
PLR	186.53 ± 105.94	129.30 ± 80.11	191.18 ± 103.99	.002

NLR = neutrophil-lymphocyte ratio, PLR = platelet-lymphocyte ratio, SII = systemic immune inflammation index, SIRI = systemic inflammation response index.

### 3.2. Factors associated with pneumonia

Among all included patients, a total of 163 (81.5%) had pneumonia after surgeries. The median surgical duration in patients with pneumonia was 122.5 minutes, longer than patients without pneumonia (90 minutes), *P =* .013. Among patients with pneumonia, there were 36.2% (59/163) and 28.8% (47/163) cases respectively with GCS score < 11 and intraventricular hemorrhage, more than patients without pneumonia, *P* < .05. There were no significant differences between patients with pneumonia and without pneumonia in gender, age and combination of lung diseases (*P >* .05). Among patients with pneumonia, the preoperative neutrophil count was 8.27 ± 5.80 × 10^9^/L, significantly higher than patients without pneumonia ([4.95 ± 3.34] × 10^9^/L), *P <* .001. Compared with patients without pneumonia, patients with pneumonia had a significantly higher SIRI ([4.57 ± 6.99] × 10^9^/L vs [2.10 ± 2.61] × 10^9^/L, *P* < .001), SII ([1451.55 ± 1973.28] × 10^9^/L vs [732.88 ± 571.87] × 10^9^/L, *P* = .001) and NLR (8.77 ± 11.66 vs 4.62 ± 3.78, *P =* .001). There were no significant differences in preoperative levels of lymphocytes, platelets and PLR, *P* > .05 (Table 2).

On the 1st postoperative day, NLR level was 12.59 ± 9.29, PLR level was 188.34 ± 148.23, SIRI level was 7.82 ± 9.97 × 10^9^/L, and the SII level was 1741.64 ± 1548.52 × 10^9^/L in all patients. The NLR level on the 1st postoperative day in patients with pneumonia was 13.12 ± 9.92, higher than patients without pneumonia (10.26 ± 4.38), *P* = .039. There were no significant differences in other inflammatory indicators, including levels of neutrophils, lymphocytes, platelets, SIRI, SII and PLR, *P >* .05 (Table 2).

On the 3rd postoperative day, NLR level was 6.18 ± 5.65, PLR level was 186.53 ± 105.94, SIRI level was 4.77 ± 6.25 × 10^9^/L, and the SII level was 1181.57 ± 1098.44 × 10^9^/L. The neutrophil count on the 3rd postoperative day in patients with pneumonia was 7.39 ± 3.89 × 10^9^/L, higher than patients without pneumonia (5.25 ± 2.44 × 10^9^/L), *P* = .007. The lymphocyte counts in these patients were 1.07 ± 0.62 × 10^9^/L and 1.33 ± 0.56 × 10^9^/L respectively, *P =* .038. Among patients with pneumonia, the SIRI level was 5.28 ± 7.09 × 10^9^/L, higher than patients with without pneumonia (3.27 ± 3.24 × 10^9^/L), *P =* .003. The SII level was 1294.18 ± 1110.95 × 10^9^/L, significantly higher, *P <* .001. Compared with patients without pneumonia, patients with pneumonia also had significantly higher NLR (6.83 ± 6.09 vs 4.36 ± 2.36, *P <* .001) and PLR level (191.18 ± 103.99 vs 129.30 ± 80.11, *P =* .002) (Table 2).

By ROC analysis, the cutoff value of surgical duration for predicting pneumonia was 132.5 minutes. The cutoff values of preoperative and the 3rd postoperative SIRI level were 2.32 × 10^9^/L and 3.94 × 10^9^/L respectively. The cutoff values of preoperative and the 3rd postoperative SII level were 1254.27 × 10^9^/L and 1172.48 × 10^9^/L respectively. The remaining cutoff values were shown in Supplemental Table 1, http://links.lww.com/MD/K445. By multivariate logistic regression analysis, there were no inflammatory indicators at different times associated with pneumonia in cerebral hemorrhage patients.

### 3.3. Factors associated with severe pneumonia

Among 163 patients with pneumonia, 60 (36.8%) cases were evaluated as severe pneumonia, and 103 (63.2%) cases were evaluated as non-severe pneumonia. Among patients with severe pneumonia, there were 48 males and 12 females. The median age was 64 years old. There were 19 cases with lung diseases. The median surgical duration in patients with severe pneumonia was 160 minutes, significantly longer than patients with non-severe pneumonia (100 minutes), *P <* .001. Among patients with severe pneumonia, 70% (42/60) had a GCS score < 11, significantly more than patients with non-severe pneumonia, *P <* .001 (Table 1). The preoperative platelet count was 167 ± 69 × 10^9^/L in these patients with severe pneumonia, lower than patients with non-severe pneumonia (193 ± 73 × 10^9^/L, *P =* .022). The PLR levels in patients with severe pneumonia, non-severe pneumonia were 136.85 ± 145.47 and 181.12 ± 162.71 respectively (*P =* .035). There were no significant differences in levels of neutrophils, lymphocytes, monocytes, SII, SIRI and NLR in these patients (*P >* .05) (Table 3).

**Table 3:** Levels of inflammatory indicators in patients with severe pneumonia and non-severe pneumonia.

	Severe pneumonia (n = 60)	Non-severe pneumonia (n = 103)	*P* value
On the preoperative day			
Neutrophils (×10^9^/L)	9.94 ± 5.29	8.77 ± 4.28	.123
Lymphocytes (×10^9^/L)	0.97 ± 1.10	1.01 ± 0.88	.814
Monocytes (×10^9^/L)	0.58 ± 0.39	0.54 ± 0.35	.115
Platelets (×10^9^/L)	167 ± 69	193 ± 73	.022
SIRI (×10^9^/L)	5.44 ± 10.02	4.26 ± 5.52	.167
SII (×10^9^/L)	1137.00 ± 2029.69	1578.84 ± 1957.20	.404
NLR	8.82 ± 11.81	8.77 ± 10.95	.615
PLR	136.85 ± 145.47	181.12 ± 162.71	.035
On the 1st postoperative day			
Neutrophils (×10^9^/L)	9.74 ± 3.74	9.28 ± 4.63	.256
Lymphocytes (×10^9^/L)	0.65 ± 0.38	0.83 ± 0.51	<.001
Monocytes (×10^9^/L)	0.77 ± 0.44	0.65 ± 0.46	.331
Platelets (×10^9^/L)	135 ± 58	174 ± 62	<.001
SIRI (×10^9^/L)	10.89 ± 12.10	7.14 ± 9.76	.003
SII (×10^9^/L)	1854.73 ± 2197.51	1708.50 ± 1558.49	.610
NLR	15.22 ± 10.77	11.35 ± 9.52	<.001
PLR	188.37 ± 158.52	191.23 ± 153.78	.674
On the 3rd postoperative day			
Neutrophils (×10^9^/L)	8.40 ± 3.10	6.71 ± 2.61	.001
Lymphocytes (×10^9^/L)	0.93 ± 0.56	1.11 ± 0.71	.006
Monocytes (×10^9^/L)	0.84 ± 0.52	0.71 ± 0.35	.137
Platelets (×10^9^/L)	190 ± 93	211 ± 83	.182
SIRI (×10^9^/L)	7.98 ± 7.46	4.10 ± 3.74	<.001
SII (×10^9^/L)	1359.44 ± 1290.59	1112.49 ± 1019.71	.016
NLR	9.44 ± 7.38	5.67 ± 4.50	<.001
PLR	193.22 ± 123.38	190.99 ± 90.40	.392

NLR = neutrophil-lymphocyte ratio, PLR = platelet-lymphocyte ratio, SII = systemic immune inflammation index, SIRI = systemic inflammation response index.

On the 1st postoperative day, the lymphocyte count was 0.65 ± 0.38 × 10^9^/L in patients with severe pneumonia, significantly lower than patients with non-severe pneumonia (0.83 ± 0.51 × 10^9^/L), *P <* .001. The platelet count was also significantly lower in patients with severe pneumonia (135 ± 58 × 10^9^/L), *P <* .001. Compared with patients with non-severe pneumonia, the levels of SIRI (10.89 ± 12.10 × 10^9^/L vs 7.14 ± 9.76 × 10^9^/L, *P =* .003) and NLR (15.22 ± 10.77 vs 11.35 ± 9.52, *P <* .001) were significantly higher. No significant differences were showed in levels of neutrophils, monocytes, SII and PLR (*P >* .05) (Table 3).

On the 3rd postoperative day, the neutrophil count in patients with severe pneumonia was 8.40 ± 3.10 × 10^9^/L, higher than patients with non-severe pneumonia (6.71 ± 2.61 × 10^9^/L), *P =* .001. The lymphocyte count was 0.93 ± 0.56 × 10^9^/L, significantly lower (1.11 ± 0.71 × 10^9^/L), *P =* .006. Patients with severe pneumonia had significantly higher SIRI level (7.98 ± 7.46 × 10^9^/L vs 4.10 ± 3.74 × 10^9^/L, *P <* .001) and SII level (1359.44 ± 1290.59 × 10^9^/L vs 1112.49 ± 1019.71 × 10^9^/L, *P =* .016). There were no significant differences in levels of monocytes, platelets and PLR between patients with severe pneumonia and patients with non-severe pneumonia (*P >* .05) (Table 3).

**Figure 2.**
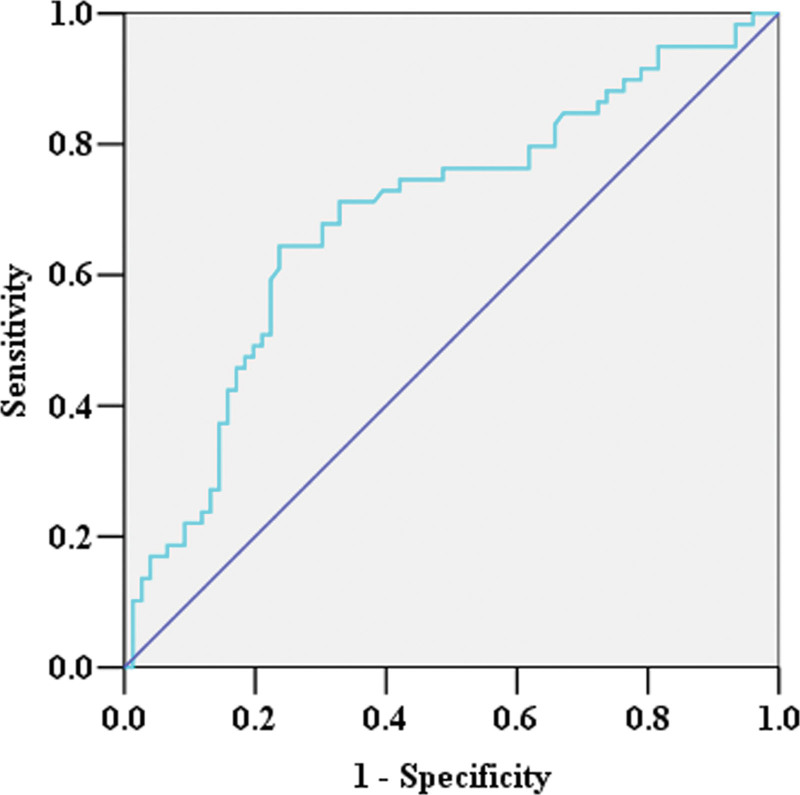
The result of ROC analysis of SIRI level on the 3rd postoperative day in patients with severe pneumonia. The cutoff value of the 3rd postoperative SIRI level for predicting severe pneumonia was 6.5 × 10^9^/L. The area under the curve was 0.692 (95% CI, 0.6–0.783, *P <* .001). The sensitivity was 64.4%, and the specificity was 76.3%. ROC = receiver operating characteristic curve, SIRI = Systemic inflammation response index.

**Figure 3.**
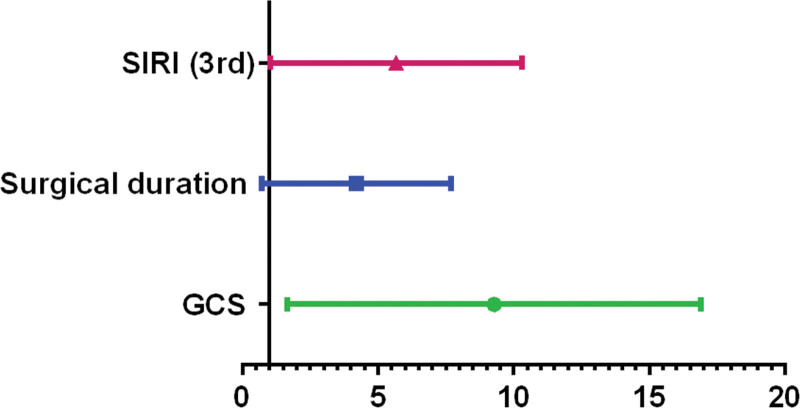
The result of multivariate logistic analysis. The result of multivariate analysis showed that SIRI level on the 3rd postoperative day (>6.5 × 10^9^/L) was associated with severe pneumonia in cerebral hemorrhage patients (OR: 4.409, 95% CI: 1.799–10.806, *P =* .001). Other factors associated with severe pneumonia included surgical duration (>152.5 min) (OR: 3.242, 95% CI: 1.300–8.085, *P =* .012) and GCS (<11) (OR: 7.207, 95% CI: 2.932–17.714, *P <* .001). GCS = Glasgow coma scale, SIRI = Systemic inflammation response index.

By ROC analysis, the cutoff value of surgical duration for predicting severe pneumonia in cerebral hemorrhage patients was 152.5 minutes. The cutoff values of preoperative and the 3rd postoperative NLR levels were 13.17 and 8.73 respectively. The cutoff value of the 3rd postoperative SIRI level was 6.5 × 10^9^/L (Fig. 2). The remaining cutoff values were shown in Supplemental Table 1, http://links.lww.com/MD/K445. The results of multivariate analysis showed that SIRI level on the 3rd postoperative day (>6.5 × 10^9^/L) was associated with severe pneumonia in cerebral hemorrhage patients (OR: 4.409, 95% CI: 1.799–10.806, *P =* .001). Other factors associated with severe pneumonia included surgical duration (>152.5 minutes) (OR: 3.242, 95% CI: 1.300–8.085, *P* = .012) and GCS (<11) (OR: 7.207, 95% CI: 2.932–17.714, *P <* .001) (Fig. 3). The positive predictive value of the model for severe pneumonia in cerebral hemorrhage patients was 78.43% (40/51), the negative predictive value was 77.92% (60/77), the sensitivity was 70.18% (40/57), and the specificity was 84.51% (60/71).

## 4. Discussions

The association between inflammatory factor and pneumonia in cerebral hemorrhage patients has been a hot topic in recent years. In our study, we explored the associations of inflammatory factors from blood cell counts on preoperative, 1st postoperative and 3rd postoperative days with pneumonia and severe pneumonia in cerebral hemorrhage patients.

The disruption of the balance between neutrophils and lymphocytes could exacerbate inflammatory responses. NLR is the ratio of neutrophils to lymphocytes, which is a new inflammatory indicator and with simplicity and convenience. Among patients with acute cerebral infarction, the NLR levels varied among patients with different infarction areas.^[14]^ Among patients with acute cerebral hemorrhage, the level of NLR was significantly higher in dead patients than in survivors.^[15]^ The results of 1 study in mice model of cerebral hemorrhage by injecting autologous blood into striatum showed that 36.4% cerebral hemorrhage mice had spontaneous pneumonia and/or bloodstream infections, while sham surgery mice had no infectious complications.^[16]^ In 1 study involving 146 patients with acute pancreatitis, a significant increase of NLR level at admission was associated with severe acute pancreatitis.^[17]^ The levels of NLR possibly varied at different times of cerebral hemorrhage. There were few studies on the associations between postoperative pneumonia and NLR levels at different times. In the study by Wang RH,^[10]^ the result showed that NLR at admission was the best predictive factor for stroke associated pneumonia. The univariate analysis results in our study found that NLR levels on the 1st and 3rd postoperative days were associated with pneumonia in cerebral hemorrhage patients. However, the multivariate analysis did not find the associations between NLR at different times and pneumonia. Different NLR levels were possibly consistent with the severity of disease. In the study of endocarditis patients, NLR > 5.035 was associated with severe sepsis.^[18]^ Our study also explored the associations between NLR at different times with severe pneumonia in cerebral hemorrhage patients. Unfortunately, only the univariate analysis showed a association between NLR level on the 1st postoperative day with severe pneumonia.

PLR was the ratio of platelet to lymphocyte. In malignant tumor patients, PLR level was possibly associated with the prognosis and treatment response to anti-tumor drugs. In endometrial cancer patients, PLR ≥ 134.95 was associated with invasion of myometrium ≥50%.^[19]^ In advanced non-small cell lung cancer patients, higher PLR level was an independent risk factor associated with the first-line chemotherapy efficacy and clinical prognosis.^[20]^ In addition to tumors, the results of other studies also confirmed the association between PLR level and infection. In patients with urinary tract infection, the PLR level was 176.645 ± 110.051, significantly higher than patients without urinary tract infection (121.945 ± 53.735).^[21]^ In patients with COVID-19, PLR level was the only factor associated with mortality.^[22]^ Our study explored the association between PLR level and postoperative pneumonia in cerebral hemorrhage patients. The univariate analysis results showed a significant association between PLR level on the 3rd postoperative day and pneumonia in cerebral hemorrhage patients. But the multivariate analysis result did not find this significant association. In the study by Ravindra R. et al, the PLR level in patients with severe infection was significantly higher than patients with mild infection.^[23]^ Our study explored the associations between PLR levels at different times and severe pneumonia in cerebral hemorrhage patients. The results of univariate analysis showed a significant difference in PLR levels between severe and non-severe pneumonia patients. However, the multivariate analysis did not show any association.

There were also some studies showing a association between SII level and prognosis. Higher SII level (>390 × 10^9^/L) was a powerful indicator for tumor differentiation and 1-year survival rate in newly diagnosed solid tumor patients.^[24]^ In pancreatic cancer and small cell lung cancer patients, significant increase of SII level was associated with a shorter overall survival.^[25,26]^ Among 18,609 stroke patients included in 19 retrospective studies, higher SII level was significantly associated with poor outcome.^[27]^ Among 362 ischemic stroke patients, SII was an independent risk factor for stroke severity.^[28]^ There were few studies on the associations between SII levels at different times and pneumonia in cerebral hemorrhage patients. Our study explored these associations, only the univariate analysis results showed that preoperative and postoperative levels of SII were associated with pneumonia and severe pneumonia.

Systemic inflammatory response index (SIRI) was based on counts of neutrophils, monocytes and lymphocytes. It was also showed to be associated with the prognosis of patients with malignant tumors. In breast cancer patients, lower SIRI was associated with higher overall survival when compared with higher SIRI.^[29]^ In gallbladder cancer patients, SIRI level was also an independent prognostic indicator.^[30]^ In pancreatic cancer patients, cases with SIRI ≥ 2.3 × 10^9^/L were more likely to benefit from mFOLFIRINOX treatment.^[31]^ Stroke is a disease with high mortality. The results of 1 study including 2450 stroke patients showed that higher SIRI level was associated with higher all-cause mortality. The increase in SIRI was associated with higher mortality and stroke severity, as well as higher sepsis risk.^[32]^ In study by Yu T. et al,^[9]^ the results showed an association between higher SIRI level at admission and pneumonia in cerebral hemorrhage patients. In our study, the result of univariate analysis confirmed this association. We also confirmed an association between SIRI level on the 3rd postoperative day and pneumonia in these patients by univariate analysis. But the multivariate analysis did not confirm these results. Our study firstly explored the association between SIRI level and severe pneumonia in cerebral hemorrhage patients. Both univariate and multivariate analyses confirmed an association between SIRI level on the 3rd postoperative day and severe pneumonia in cerebral hemorrhage patients.

Our study also had several limitations. First, the preoperative levels of inflammatory factors in most patients were on the 1st preoperative day, in a very few patients were on the 2nd preoperative day, which possibly resulted a bias. Second, the study only investigated the associations of blood cell counts and related indicators with pneumonia in cerebral hemorrhage patients, but did not investigate other inflammatory indicators including C-reactive protein, interleukin-6 and other inflammatory factors. Third, the sample size was not big enough, and we will expand the sample size for the next step.

## 5. Conclusion

SIRI was possibly a superior predictor for severe pneumonia in cerebral hemorrhage patients compared with other inflammatory indicators. On the one hand, we intend to validate the cutoff value of SIRI for predicting severe pneumonia in larger samples and multicenter studies. On the other hand, we also intend to use this index to guide the choice of antibacterial drugs in order to better benefit patients.

## Acknowledgments

We are thankful to all the medical staff and statistician in the department of Neurosurgery, The First Affiliated Hospital of Yangtze University.

## Author contributions

**Conceptualization:** Yongfeng Zhao, Xian Wang, Yuan Yao.

**Data curation:** Xian Wang.

**Formal analysis:** Yongfeng Zhao.

**Methodology:** Hongbo Ren, Yuan Yao.

**Project administration:** Xian Wang, Yuan Yao.

**Writing – original draft:** Yongfeng Zhao, Xian Wang.

**Writing – review & editing:** Xian Wang, Yuan Yao.

## Supplementary Material


