# Supplementary material for: Systemic inflammation response index (SIRI) on the 3rd postoperative day are associated with severe pneumonia in cerebral hemorrhage patients: A single-center retrospective study
Source: Medicine (Baltimore). 2023 Oct 27;102(43):e35587. doi: 10.1097/MD.0000000000035587 (PMC10615502; doi:10.1097/MD.0000000000035587)
Supplement: Supplementary file 1 [file medi-102-e35587-s001.docx]

|  | Area | Sensitivity | Specificity | Youden index | Cut-off value | P |
| --- | --- | --- | --- | --- | --- | --- |
| For Pneumonia | | | | | | |
| Preoperative Neutrophils | 0.736 | 71.20% | 72.20% | 0.434 | 13.165 | < .001 |
| Preoperative Monocytes | 0.632 | 52.80% | 74.30% | 0.271 | 0.545 | .014 |
| Preoperative SIRI | 0.697 | 73.00% | 65.70% | 0.387 | 2.315 | < .001 |
| Preoperative SII | 0.680 | 56.40% | 80.00% | 0.364 | 1254.27 | .001 |
| Preoperative NLR | 0.685 | 59.50% | 77.10% | 0.366 | 6.955 | .001 |
| 1st Postoperative NLR | 0.627 | 43.30% | 88.50% | 0.318 | 14.01 | .039 |
| 3rd Postoperative Lymphocytes | 0.647 | 68.40% | 61.50% | 0.299 | 1.195 | .038 |
| 3rd Postoperative SIRI | 0.712 | 64.40% | 73.70% | 0.381 | 3.94 | .003 |
| 3rd Postoperative SII | 0.794 | 57.80% | 100.00% | 0.578 | 1172.48 | < .001 |
| 3rd Postoperative NLR | 0.747 | 64.40% | 89.50% | 0.539 | 5.58 | < .001 |
| 3rd Postoperative PLR | 0.715 | 78.50% | 57.90% | 0.364 | 131.93 | .002 |
| For Severe Pneumonia | | | | | | |
| Preoperative Platelets | 0.405 | 100.00% | 1.90% | 0.019 | 42 | .044 |
| Preoperative PLR | 0.401 | 10.00% | 92.20% | 0.022 | 389.05 | .035 |
| 1st Postoperative Lymphocytes | 0.678 | 87.90% | 40.70% | 0.286 | 0.535 | < .001 |
| 1st Postoperative Platelets | 0.670 | 85.70% | 42.40% | 0.281 | 115.5 | < .001 |
| 1st Postoperative SIRI | 0.643 | 54.20% | 69.20% | 0.234 | 10.38 | .003 |
| 1st Postoperative NLR | 0.686 | 71.20% | 64.80% | 0.36 | 13.165 | < .001 |
| 3rd Postoperative Neutrophils | 0.651 | 59.30% | 67.10% | 0.264 | 7.8 | .003 |
| 3rd Postoperative Lymphocytes | 0.639 | 31.60% | 93.20% | 0.248 | 1.445 | .006 |
| 3rd Postoperative SIRI | 0.692 | 64.40% | 76.30% | 0.407 | 6.5 | < .001 |
| 3rd Postoperative SII | 0.622 | 74.60% | 50.00% | 0.246 | 1093.575 | .016 |
| 3rd Postoperative NLR | 0.721 | 57.60% | 78.90% | 0.365 | 8.73 | < .001 |
| Surgical duration | 0.692 | 59.30% | 76.70% | 0.36 | 152.5 | < .001 |

**Supplemental Table 1.** The cut-off values of factors for predicting pneumonia and severe in cerebral hemorrhage patients

SII = Systemic immune inflammation index, SIRI = Systemic inflammation response index, NLR = Neutrophil-lymphocyte ratio, PLR = Platelet-lymphocyte ratio.
